# Cancer Prevention with Molecular Targeted Therapies

**DOI:** 10.3390/ijms23158429

**Published:** 2022-07-29

**Authors:** Laura Paleari

**Affiliations:** Research, Innovation and HTA Unit, A.Li.Sa., Liguria Health Authority, 16121 Genoa, Italy; laura.paleari@alisa.liguria.it; Tel.: +39-010-5484243

Today, the oncologist is like a detective of the human body who, instead of a magnifying glass, uses the new tools of molecular pathology to search not only for genes or molecular targets, to be targeted with innovative anticancer therapies, but also molecular alterations that allow the identification of population groups at risk of developing tumors for preventive purposes. This is the precision oncology thanks to which it is possible today to aim at treatments but also at personalized cancer prevention, based on precision-medicine models, through the identification of specific genomic determinants linked to an increased risk of developing cancer. This area includes a series of interventions to identify cancer at an early stage or to avoid the onset of the disease. A paradigmatic example is represented by the BRCA mutational status in breast cancer: women with a mutation in the BRCA gene, which represents a risk factor for breast cancer, may be offered more frequent breast-screening programs, which are part of secondary prevention, or treatment with aromatase inhibitors or antiestrogens to enhance primary prevention [1]. Molecular pathology is a cornerstone of precision oncology, and today, it is necessary to learn to study not only the single alterations but also the overall modifications of the cellular signal transduction pathways. In this way, the molecular pathologist can provide the clinician with crucial information to drive the therapeutic choice. The so-called histological model, which has long governed clinical research in oncology and clinical practice, is now flanked by the molecular model [2,3,4]. In this approach, the starting point is represented by the organ from which the neoplasia originates, followed by the histological examination, the identification of any molecular alterations and the choice of the drug, through a path of selecting patients who are more likely to respond to treatment. The histological model has been exceeded by the agnostic one, in which oncological therapies are chosen based on specific genomic alterations or particular molecular aspects that may also be present in various tumors, which represent the cellular target. Interestingly, the mutational model also includes the microbiota, which represents the set of billions of microorganisms that live in the body, providing essential support for our life [5]. Targeted anticancer therapies involve the use of drugs that block the growth and spread of cancer cells by interfering with molecules involved in tumor progression, called “molecular targets”. Targeted anticancer therapies differ from classic chemotherapy in several aspects: (i) they act on specific molecular targets of cancer cells, while most chemotherapies act on all cells that reproduce rapidly, both normal and cancerous; (ii) they block the proliferation of tumor cells (cytostatic), while chemotherapy drugs kill tumor cells (cytotoxic); (iii) they interact specifically with their target, while many chemotherapies are identified on the basis of their cytotoxic capacity. Targeted anticancer therapies are currently at the heart of the development of many anticancer drugs including hormone therapies, signal transduction inhibitors, gene expression modulators, apoptosis inducers, angiogenesis inhibitors, immunotherapies and toxin-releasing compounds. It is important to note that targeted cancer therapies have some limits concerning the possibility of acquired resistance to the treatment. For this reason, targeted anticancer therapies work best in combination. For example, a recent study found that using two drugs in melanoma with the BRAF V600E mutation slows the development of resistance and disease progression more than using just one drug alone [6]. Additionally, it is possible to use molecular targeted therapy in combination with one or more traditional chemotherapy drug. For example, therapy using pertuzumab and trastuzumab was used in combination with docetaxel, a traditional chemotherapy drug, to treat women with HER-2 protein metastatic breast cancer [7]. Another limitation of targeted therapy is that drugs for some identified targets are difficult to develop due to the structure of the target and/or the way its function is regulated by the cell. An example is represented by Ras, a family of proteins involved in the transmission of signals within the cell, which are mutated in a quarter of all cancers. To date, it has not been possible to develop Ras signaling inhibitors. However, new pharmacological approaches show promising preliminary results for the future [8].

The purpose of this Special Issue is to highlight the importance of research aiming at discovering new and more informative methods of diagnostic molecular testing, targeted therapies mainly in early-stage disease, and future directions for precision oncology approaches to understand the tumor evolution and the possible related therapeutic resistance.
